# SENOLYTIC DRUGS: DISCOVERY, TRANSLATION, AND APPLICATION

**DOI:** 10.1093/geroni/igz038.2730

**Published:** 2019-11-08

**Authors:** Nathan LeBrasseur

**Affiliations:** Robert and Arlene Kogod Center on Aging, Mayo Clinic, Rochester, Minnesota, United States

## Abstract

Diverse forms of molecular and cellular stress trigger senescence, a state of growth arrest in proliferation-competent cells that is often accompanied by a robust secretory phenotype. Senescent cell burden increases with age in multiple tissues and, plausibly, contributes to the pathogenesis of age-related diseases and geriatric syndromes. Discovery science efforts have identified druggable targets in senescent cells, including key nodes in anti-apoptosis pathways, that distinguish them from non-senescent counterparts and enable pharmacological approaches for their selective elimination. The therapeutic potential of senolytic interventions to improve health- and lifespan has been supported by translational research studies, including murine models of aging, atherosclerosis, osteoporosis, neurodegeneration, pulmonary fibrosis, and frailty. These studies have informed the design of first-in-human clinical trials of senolytic drugs, which have recently begun. The objective of this lecture is to highlight both the progress and challenges of advancing interventions targeting senescent cells from bench to bedside.

